# Supplementary figures and images for: Hibiscetin attenuates lipopolysaccharide-evoked memory impairment by inhibiting BDNF/caspase-3/NF-κB pathway in rodents
Source: PeerJ. 2024 Jan 31;12:e16795. doi: 10.7717/peerj.16795 (PMC10838095; doi:10.7717/peerj.16795)

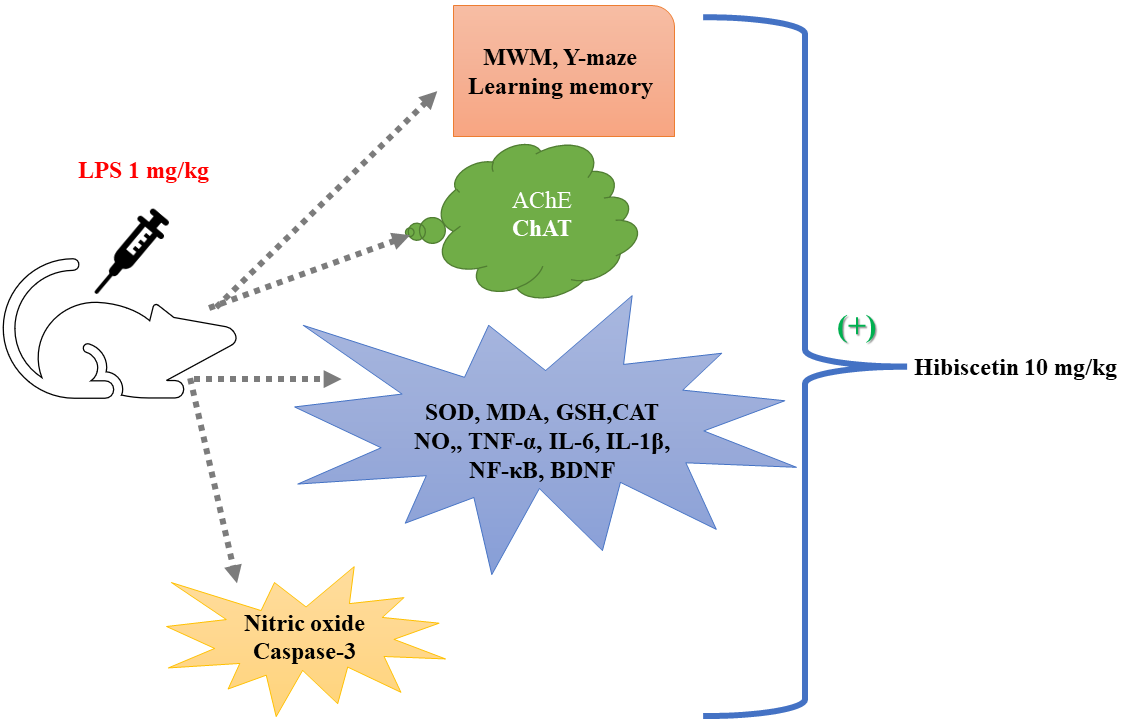

Supplement: Supplemental Information 1 [file peerj-12-16795-s001.tif]
